# Uncontrolled hypertension increases risk of all-cause and cardiovascular disease mortality in US adults: the NHANES III Linked Mortality Study

**DOI:** 10.1038/s41598-018-27377-2

**Published:** 2018-06-20

**Authors:** Donghao Zhou, Bo Xi, Min Zhao, Liang Wang, Sreenivas P. Veeranki

**Affiliations:** 1 Department of Endocrinology, Linyi People’s Hospital, 276003 Linyi, China; 2 Department of Epidemiology, School of Public Health, Shandong University, Jinan, China; 3 Department of Nutrition and Food Hygiene, School of Public Health, Shandong University, Jinan, China; 4 Department of Biostatistics and Epidemiology, College of Public Health, East Tennessee State University, Johnson City, Tennessee USA; 5 Department of Preventive Medicine and Community Health, University of Texas Medical Branch, Galveston, Texas USA

## Abstract

Clinical trials had provided evidence for the benefit effect of antihypertensive treatments in preventing future cardiovascular disease (CVD) events; however, the association between hypertension, whether treated/untreated or controlled/uncontrolled and risk of mortality in US population has been poorly understood. A total of 13,947 US adults aged ≥18 years enrolled in the Third National Health and Nutrition Examination Survey (1988–1994) were used to conduct this study. Mortality outcome events included all-cause, CVD-specific, heart disease-specific and cerebrovascular disease-specific deaths, which were obtained from linked 2011 National Death Index (NDI) files. During a median follow-up of 19.1 years, there were 3,550 all-cause deaths, including 1,027 CVD deaths. Compared with normotensives, treated but uncontrolled hypertensive patients were at higher risk of all-cause (HR = 1.62, 95%CI = 1.35–1.95), CVD-specific (HR = 2.23, 95%CI = 1.66–2.99), heart disease-specific (HR = 2.19, 95%CI = 1.57–3.05) and cerebrovascular disease-specific (HR = 3.01, 95%CI = 1.91–4.73) mortality. Additionally, untreated hypertensive patients had increased risk of all-cause (HR = 1.40, 95%CI = 1.21–1.62), CVD-specific (HR = 1.77, 95%CI = 1.34–2.35), heart disease-specific (HR = 1.69, 95%CI = 1.23–2.32) and cerebrovascular disease-specific death (HR = 2.53, 95%CI = 1.52–4.23). No significant differences were identified between normotensives, and treated and controlled hypertensives (all *p* > 0.05). Our study findings emphasize the benefit of secondary prevention in hypertensive patients and primary prevention in general population to prevent risk of mortality later in life.

## Introduction

Cardiovascular disease (CVD) is the leading cause of deaths worldwide, accounting for 17.3 million deaths per year which is expected to increase to >23.6 million by 2030^1^. Hypertension is known to be the major risk factor for global CVD morbidity and mortality, with an estimated half of the CVD events attributed to it^2^. Thus, it is important to prevent, treat and control hypertension to reduce the risk of CVD events and related healthcare burden.

The Eighth Joint National Committee (JNC 8) guidelines recommend the systolic/diastolic blood pressure (SBP/DBP) to be under 140/90 mmHg for all treated hypertensive individuals aged <60 years and 150/90 mmHg for those aged ≥60 years^3^. Abundant evidence from Randomized Controlled Trials (RCTs) has shown the benefit effect of antihypertensive drug treatment in reducing the risk of CVD events in hypertensive patients^4^. However, most RCTs are subject to several limitations, which include: (1) External validity of the results^5^, and (2) Less duration of follow-up duration to study the long-term CVD outcomes, especially in young patients^6^. To address these limitations, several observational studies have examined the effect of controlled or uncontrolled treated hypertension on the risk of CVD events compared to normotensive subjects, however results were inconsistent^7–12^. Some studies reported that hypertensive patients with controlled BP were still at higher risk of CVD mortality than normotensive ones^7–9,11^, while other found no such evidence^10,12^.

In the current study, we investigated the association of controlled and uncontrolled hypertension, and the role of treatment, with the risk of all-cause and CVD mortality using a prospective cohort of a nationally representative sample of US adults. To investigate the disparities, we also examined the relationship by sex, age and race/ethnicity.

## Results

### Baseline characteristics

The final study sample consisted of 13,947 subjects (women: 50.8%, age range: 18–90 years). Approximately 26% were found to be hypertensive at baseline. Among them, 20.4% were treated and controlled, 27.1% were treated but uncontrolled, and 52.5% were untreated. Table 1 shows the baseline characteristics of study participants, and presents key differences in baseline characteristics by study exposure categories. There were significant differences in all baseline characteristics between four groups by hypertension status (*p* < 0.001). Among hypertensive participants, treated but uncontrolled hypertensive participants were found to be different from other two hypertensive groups. Approximately 17% of treated but uncontrolled hypertensive participants were non-Hispanic blacks compared to 13.8% and 13.4% of those in the treated and controlled group, and untreated group, respectively. Similarly, the average BMI, SBP and TC were significantly higher than other two groups.

**Table 1:** Baseline characteristics of study population.

	Overall	Hypertension status				*p* value
		Normal	Treated & controlled	Treated & uncontrolled	Untreated	
N (%)	13,947 (100)	10,300 (73.9)	743 (5.3)	989 (7.1)	1,915 (13.7)	
Age group, %						<0.001
18–39 yrs	52.2	61.5	11.9	5.5	20.6	
40–59 yrs	31.4	29.7	46.3	34.1	36.0	
≥60 yrs	16.5	8.8	41.9	60.4	43.5	
Women, %	50.8	50.7	63.9	58.8	42.6	<0.001
Race/ethnicity, %						<0.001
Non-Hispanic white	75.2	74.9	78.7	75.8	75.4	
Non-Hispanic black	11.0	10.2	13.8	16.6	13.4	
Mexican-American	5.5	6.0	2.3	3.2	4.5	
Other	8.3	8.9	5.1	4.5	6.6	
Education level, %						<0.001
<12 yrs	23.6	21.4	30.7	39.2	30.3	
12–15 yrs	55.7	56.6	55.0	45.9	53.5	
≥16 yrs	20.7	22.0	14.2	14.9	16.3	
BMI, kg/m^2^, (mean ± SE)	26.3 ± 0.1	25.7 ± 0.1	29.8 ± 0.5	30.0 ± 0.3	28.4 ± 0.2	<0.001
SBP, mmHg, (mean ± SE)	120.8 ± 0.4	115.0 ± 0.2	125.1 ± 0.6	153.7 ± 0.7	147.7 ± 0.5	<0.001
DBP, mmHg, (mean ± SE)	74.0 ± 0.2	71.8 ± 0.2	76.2 ± 0.4	83.0 ± 0.6	85.7 ± 0.4	<0.001
Smoking status, %						<0.001
Never	44.8	45.4	41.5	46.1	41.8	
Former	24.1	21.7	37.7	36.7	30.8	
Current	31.0	32.9	20.9	17.1	27.3	
Alcohol intake, %						<0.001
Never	56.0	54.1	68.4	72.1	57.0	
1–2 drinks/wk	20.6	22.0	16.3	9.8	17.1	
≥3 drinks/wk	23.4	23.9	15.3	18.1	25.9	
TC, mg/dl, (mean ± SE)	200.7 ± 0.7	195.0 ± 0.8	223.6 ± 1.9	227.5 ± 1.7	222.1 ± 1.4	<0.001
Lipid lowering medications use,%	2.1	1.1	11.7	9.0	2.4	<0.001
Diagnosed diabetes, %	4.2	2.8	13.6	13.6	6.1	<0.001

BMI means body mass index; DBP, diastolic blood pressure; SBP means systolic blood pressure; TC means total cholesterol.

### Association of hypertension status with all-cause and CVD-specific mortality

**Figure 1:**
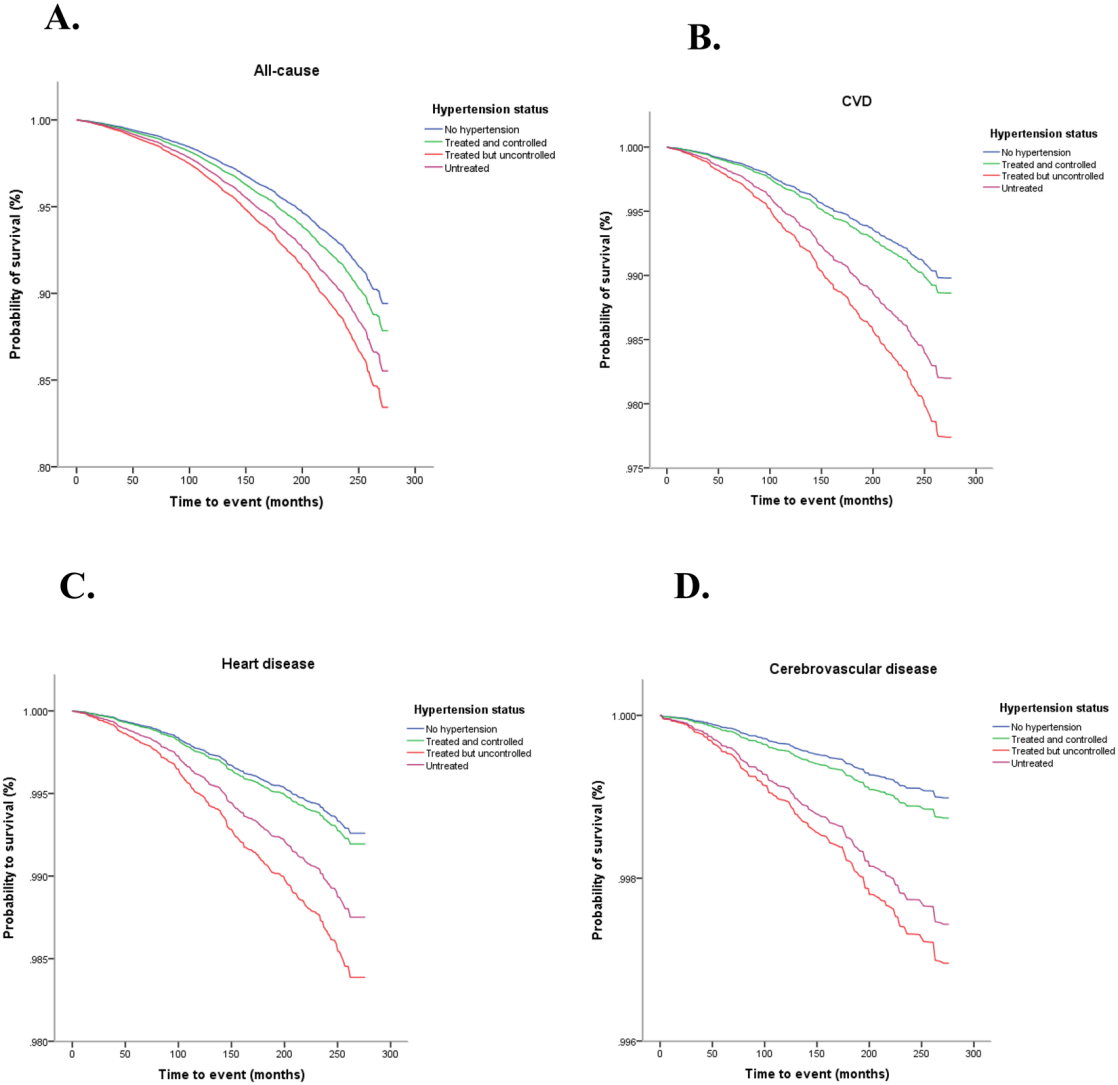
Kaplan-Meier survival estimates for (**A**) all-cause mortality, (**B**) CVD-specific mortality, (**C**) heart disease-specific mortality, and (**D**) cerebrovascular disease-specific mortality among four study exposure groups.

During a median follow-up of 19.1 years (IQR 17.4–21.0), there were 3,550 all-cause deaths, including 1,027 CVD deaths (771 heart disease-specific deaths and 256 cerebrovascular disease-specific deaths). Kaplan-Meier curve analyses showed that there were statistically significant differences in survival probabilities between treated but uncontrolled hypertensive and normotensive individuals, and between untreated hypertensive and normotensive participants for all outcome events (log rank test, *p* < 0.05 for all) (see Fig. 1A–D). However, there was no difference between treated and controlled hypertensives and normotensives (log rank test, *p* > 0.05 for all). When adjusting for potential confounding, treated but uncontrolled hypertensive participants were found to be at higher risk for all-cause (HR = 1.62, 95%CI = 1.35–1.95), CVD-specific (HR = 2.23, 95%CI = 1.66–2.99), heart disease-specific (HR = 2.19, 95%CI = 1.57–3.05) and cerebrovascular disease-specific deaths (HR = 3.01, 95%CI = 1.91–4.73) than normotensive participants. In addition, untreated hypertensive patients had increased risk of all-cause (HR = 1.40, 95%CI = 1.21–1.62), CVD-specific (HR = 1.77, 95%CI = 1.34–2.35), heart disease-specific (HR = 1.69, 95%CI = 1.23–2.32) and cerebrovascular disease-specific (HR = 2.53, 95%CI = 1.52–4.23) mortality than normotensive participants (see Table 2). Importantly, no significant associations of study outcomes were found with treated and controlled hypertensive participants (all *p* > 0.05) (see Table 2).

**Table 2:** Association of hypertension status with all-cause and CVD-specific mortality.

Hypertension status	n/ N*	All-causes			CVD			Heart disease			Cerebrovascular diseases	
		HR (95%CI)	*p* value	n/N*	HR (95%CI)	*p* value	n/N*	HR (95%CI)	*p* value	n/N*	HR (95%CI)	*p* value
Normal	1561/10300	1.00 (Reference)		357/10300	1.00 (Reference)		286/10300	1.00 (Reference)		71/10300	1.00 (Reference)	
Treated & controlled	335/743	1.16 (0.95–1.42)	0.151	91/743	1.12 (0.76–1.63)	0.565	71/743	1.09 (0.71–1.67)	0.693	20/743	1.24 (0.55–2.81)	0.593
Treated & uncontrolled	635/989	1.62 (1.35–1.95)	<0.001	240/989	2.23 (1.66–2.99)	<0.001	169/989	2.19 (1.57–3.05)	<0.001	71/989	3.01 (1.91–4.73)	<0.001
Untreated	1019/1915	1.40 (1.21–1.62)	<0.001	339/1915	1.77 (1.34–2.35)	<0.001	245/1915	1.69 (1.23–2.32)	0.002	94/1915	2.53 (1.52–4.23)	0.001

*No of events/ No of participants.

CVD, Cardiovascular Disease; HR, Hazards Ratio; CI, Confidence Interval.

All Cox proportional hazards models adjusted for sex, age, race/ethnicity, education level, smoking, alcohol intake, BMI, TC, cholesterol-lowering medication use and diagnosed diabetes.

### Subgroup analysis by sex, age group and race/ethnicity

Similar patterns of associations were found between hypertension status and risk of all-cause and CVD-specific mortality when stratified by sex (men and women), age group (<60 and ≥60 years old), and race/ethnicity (non-Hispanic White, non-Hispanic black, and Mexican- American), respectively (Supplemental Table 1). Sensitivity analyses conducted using 150/90 mmHg as cut-off to define study exposure specifically for adults aged ≥60 years revealed similar findings (Supplemental Table 2).

## Discussion

In a nationally representative sample of US adults, we found that hypertensive adults who were either untreated or treated but uncontrolled hypertension were at increased risk of all-cause and CVD-specific mortality than normotensives. However, hypertensives who were treated and controlled didn’t have increased risk of all-cause or CVD-specific mortality. No racial, age- or sex-specific disparities existed in this relationship. While our study findings address the external validity of the relationships, they also re-emphasize the benefit and importance of adequate BP control in hypertensives, as well as the primary prevention of hypertension in the general US population.

We found that treated but uncontrolled hypertensive adults had increased risk of all-cause and CVD mortality than normotensive adults. This finding can be attributed to several reasons. First, this might be due to high risk factor profile with a majority of participants in this group being found to be either older, non-Hispanic Black, obese, diabetic or had lower educational status, and less likely to use lipid-lowering drugs, as also reported in earlier studies^9,11^. However, we adjusted for these confounding factors in the regression models although the potential residual effect cannot be completely ruled out. Second, patient’s non-adherence to hypertensive drugs (e.g., inadequate doses or improper antihypertensive drug types) might explain the higher risk of mortality in treated but uncontrolled hypertensives. Previous studies have reported that non-adherence to antihypertensive medications was associated with higher rates of visit-to-visit variability of BP levels^13^, increased hospitalization and readmissions rates, increased length of hospitalization stay and subsequent poor health outcomes^14,15^. Third, the proportion of resistant hypertension might be higher in treated but uncontrolled hypertensive patients. These patients usually have higher levels of SBP and have difficulty in achieving the optimal BP levels. We did not have information about the type and number of antihypertensive medications use by study participants, but the combinations of at least three antihypertensive drugs from different drug classes should be used to control this resistant hypertension^16^. Overall, prevention efforts should be targeted at reducing the preventative risk factors, increasing antihypertensive medication adherence rates, and combination of therapeutics to effectively control BP levels among treated hypertensive patients.

An important finding in our study is that there was no significantly increased risk of mortality among hypertensives who were treated and had hypertension controlled with antihypertensive drugs. Along with primary prevention efforts such as increasing levels of physical activity, reducing smoking and limiting alcohol use, and early screening efforts, it is important that the hypertensive adults were adequately treated and carefully monitored to keep their BP levels under check^9,11^. Although we did not find any sex-, race- and age-specific disparities in the relationship, efforts should be taken to increase access to care for hypertensive adults in low-income disadvantaged communities such as those residing in the CVD or stroke-belt in the southeastern United States^17^.

One previous publication by Lanti *et al*. showed that compared to non-hypertensive individuals, controlled hypertensive patients were not at increased risk of CVD (HR = 1.03, 95%CI = 0.58–1.82), whereas uncontrolled and untreated hypertensive patients were both at higher risk of CVD (uncontrolled: HR = 2.04, 95%CI = 1.46–2.85; untreated: HR = 1.95, 95%CI = 1.34–2.29), after adjustment for sufficient confounders including sex, age, cigarette smoking, heart rate, BMI, fasting blood glucose, TC, HDL-C, serum uric acid, eGFR, urine creatinine and organ damage score^18^. The findings by Lanti *et al*. are in agreement with ours although Lanti *et al*. used CVD as outcome while we used all-cause and CVD-specific morality as outcomes. Indeed, the excess risk of patients on hypertensive treatment is mainly due to uncontrolled hypertension. The Choice to separate treated hypertensive patients in controlled versus uncontrolled is clearly demonstrated in both the study by Lanti *et al*. and our study. In addition, both studies emphasize the sufficient control of BP in patients with hypertension in order to reduce long-term risk of CVD events and premature deaths.

Recently, a systematic analysis of population-based studies from 90 countries showed that the global prevalence of adult hypertension was 31.1% in 2010, and the proportions of awareness, treatment, control among treated patients, and control among all hypertensive patients were 46.5%, 36.9%, 37.1% and 13.8%, respectively^19^. These results suggest the high burden of hypertension worldwide, with the high prevalence but low control rate. An earlier study that assessed the trends using NHANES 1999–2012 found that the prevalence rate of hypertension was stable ~30%. However, the rates of awareness, treatment, control among treated patients, and control among hypertensive patients increased from 69.6%, 59.8%, 53.3%, and 32.2% in 1999–2000, respectively, to 82.0%, 74.7%, 68.9%, and 51.2% in 2011–2012, respectively^20^. Still 31% of treated hypertensives did not have their BP adequately controlled. To achieve the Healthy People 2020 hypertension control goal of 88% treated and controlled hypertension, continuous rigorous efforts are needed including obesity prevention and control, health insurance expansion to increase continuity of care, and guideline-based screening, treatment and management of hypertension and related CVD outcomes^19^.

In our study, we used <140/90 as the target to indicate adequate BP control in both young and middle-aged hypertensive patients (<60 years) and older ones (≥60 years). It is of note that the US guidelines support treating hypertensive persons aged ≥60 years to a BP goal of less than 150/90 mm Hg^3^. However, the American Heart Association and the American College of Cardiology still consider 140/90 mmHg as the optimal cut-off point for controlled hypertension for both age groups, as SBP level at 150 mmHg will increase the risk of CVD and renal outcomes compared to SBP level at 140 mmHg^21,22^. This evidence was also supported by two recent meta-analyses that demonstrated that BP when lowered to ~130/80 mmHg or less reduces CVD and death compared to 140/90 mmHg^23,24^. Additionally, the findings from the Systolic Blood Pressure Intervention Trial hints about further lowering the optimal SBP level to ~120 mmHg in older hypertensive patients with ages 75 years and older^25^. The adoption of optimal level of SBP for older adults is still under debating, so we conducted sensitivity analyses with defining our study exposure groups using SBP of 150 mmHg as optimal level in adults aged 60 years or older, and found no substantially change in the risk estimates.

Our study has important strengths to consider. First, our study included a large number of sample survey participants representative of the US national population with the long median follow-up of 19.1 years, which makes our study findings externally valid. Second, we used mean BP values from two separate occasions to define the exposure at baseline, which are consistent with the hypertension guidelines that the clinical diagnosis of hypertension required repeated measurements on at least two different occasions^3^. Previous studies have used BP values collected on a single occasion^7–12^, which might have led to misclassification bias and over/under estimation of the effect sizes. Third, we adjusted for several confounding variables for robustness in our model, although there is still a possibility for unmeasured or residual confounding. Along the same lines of strengths, our study is subject to several limitations. First, we used BP data at baseline, and the values might have changed over time which might influence the point estimates. Moreover, no information on level of adherence to antihypertensive treatment, and antihypertensive drug type and dose (level of exposure) was unavailable, which impeded us our deeper understanding on the significant association of study outcomes in treated and controlled hypertensive adults. Future research studies should be conducted to address varying study exposure and covariates, competing risks models to address possible competing risks, and to obtain additional information on drug utilization and adherence among hypertensive adults.

## Conclusions

This study suggests that untreated hypertensive adults or those treated but not having control in the US were at increased risk of all-cause, CVD-specific, heart disease-specific or cerebrovascular disease-specific mortality. An important observation is that treated and controlled hypertensives were not found to be significantly at increased risk of mortality later in life. Our study highlights the need for multi-dimensional approach at all levels of prevention including primary prevention efforts targeting early lifestyle of behavioral risk factors, secondary prevention efforts targeting treatment, adherence and management of hypertension, and tertiary prevention addressing the adverse health outcomes of hypertension later in life.

## Methods

### Study population

A prospective cohort study of US adults aged ≥18 years enrolled in the Third National Examination and Nutritional Health Survey (NHANES III) was used to conduct this study. A stratified multistage probability sampling design was employed to enroll a sample of adults representative of civilian, noninstitutionalized adults in the US^26^. Details about the NHANES III design, questionnaires and data collection can be found at http://www.cdc.gov/nchs/nhanes/nhanes3.htm. A total of 33,994 participants (including children, adolescents and adults) were interviewed in household and 30,818 were physically examined at baseline period (phase I- 1988–1991, phase II- 1991–1994). We included only adults aged ≥18 years (n = 20,050), and excluded adults who reported a previous history of cardiovascular disease or heart failure (n = 1,742), cancer (n = 1,497), reported currently being pregnant (n = 338) and those with missing covariates of interest (n = 2,191) at baseline, resulting in a final analytical sample of 13,947 subjects. In the present study, we used data from NHANES III rather than NHANES because the last data were collected since 1999–2000, and the follow-up duration is about 10 years if they are linked to 2011 death records. Thus, the shorter follow-up duration will result in limited death cases. To conduct our investigation, we used the data from the linked NHANES III-mortality data that comprised of follow-up of survey participants until December 31, 2011. The mortality data of the NHANES survey participants were obtained from the death certificate records from the National Death Index (NDI)^27^. NHANES III was approved by the institutional review board of Centers for Disease Control and Prevention, and written informed consent was obtained from each participant. All methods were carried out in accordance with relevant guidelines and regulations. All experimental protocols were approved by institutional committee of Centers for Disease Control and Prevention, and written informed consent was obtained from all participants.

### Study measures

#### Study exposure

BP was measured using a calibrated mercury sphygmomanometer with the participants in the sitting position after 5 min of rest by an interviewer at the home interview and a physician at the mobile examination center. The first Korotkoff phase was used to indicate SBP and the fifth Korotkoff phase was recorded as DBP. Up to 6 measurements from 2 separate occasions (household interview and physical examination at a mobile) were used to calculate the average BP^26^. Using a combination of BP values and drug treatment measures, we categorized our study participants into four groups: (1) Treated and controlled hypertension- use of antihypertensive medication and SBP/DBP <140/90 mmHg, (2) Treated but uncontrolled hypertension- defined as use of antihypertensive medication but SBP/DBP ≥140/90 mmHg, (3) untreated hypertension- defined as no use of antihypertensive medication and SBP/DBP ≥140/90 mmHg, and (4) normotension- defined as no use of antihypertensive medication and SBP <140/90 mmHg. Consistent with the JNC-8 guidelines^3^, we further used 150/90 mmHg as cut-offs for defining our study exposure groups for adults aged ≥60 years. We used self-reported responses of study participants to determine their use of antihypertensive medications.

#### Study outcomes

The baseline NHANES III data (1988–1994) were linked to the NDI records through 31 December, 2011 to determine the morality status using a probabilistic matching algorithm^27^. Using *International Classification of Diseases-* 10^th^ Revision (ICD-10) codes, we defined our study outcomes as (1) deaths from all causes; (2) CVD-specific mortality (including heart disease and cerebrovascular disease), (3) heart disease-specific mortality using ICD-10 codes I00-I09, I11, I13, and I20-I51, (4) cerebrovascular disease-specific mortality using ICD-10 codes I60-I69, respectively.

#### Covariates

Several additional variables that might potentially influence the relationship of hypertension with risk of mortality were included. These included (1) demographic characteristics- sex (men, women), age (18–39, 40–59, ≥60 years), race/ethnicity (non-Hispanic white, non-Hispanic black, Mexican American, other), educational level (<12, 12–15, ≥16 years), (2) lifestyle/behavior factors- body mass index (BMI), smoking status (never, former, current), and alcohol intake (never, 1–2, ≥3 drinks/week), and (3) other chronic conditions and medications: Diagnosis of diabetes (yes, no), total cholesterol (TC), and lipid medication use (yes, no). Participants’ self-reported responses were used to define these variables, except for BMI and TC defined using objective measures.

#### Statistical analysis

Baseline characteristics of the study participants were presented using proportions for categorical variables, or mean (±standard error; SE) for continuous variables. Differences between categories of hypertension (normotension, treated and controlled, treated but uncontrolled, and untreated) among participant characteristics were determined using analysis of variance for continuous variables and using chi-square for categorical variables. Kaplan-Meier survival curves were calculated for all-cause, CVD-specific, heart disease-specific and cerebrovascular disease-specific mortality stratified by categories of hypertension, and the survival curves between groups were compared using the log-rank test. Cox-proportional hazards models were constructed to estimate the association of hypertension status with all-cause, CVD-specific, heart disease-specific and cerebrovascular disease-specific mortality, with estimates adjusted for participant demographic characteristics, lifestyle/behaviors factors, and other chronic condition/treatment. Hazard ratios (HRs) and associated 95% confidence intervals (CIs) were calculated with the normotensive participants as referent category. Stratified analyses were performed by sex (men and women), age groups (<60 and ≥60 years), race/ethnicity (non-Hispanic white, non-Hispanic black, and Mexican American). All data analyses were performed by IBM SPSS statistics, version 20.0, incorporating complex sampling design (primary sampling units, sampling strata and weights) into regression modeling. Two-sided *P* < 0.05 indicates statistical significance.

## Electronic supplementary material

Supplemental Tables 1-2
