# Supplementary material for: Uncontrolled hypertension increases risk of all-cause and cardiovascular disease mortality in US adults: the NHANES III Linked Mortality Study
Source: Sci Rep. 2018 Jun 20;8:9418. doi: 10.1038/s41598-018-27377-2 (PMC6010458; doi:10.1038/s41598-018-27377-2)
Supplement: Supplementary file 1 — Supplemental Tables 1-2 [file 41598_2018_27377_MOESM1_ESM.doc]

**Uncontrolled hypertension increases risk of all-cause and cardiovascular disease mortality in US adults: the NHANES III Linked Mortality Study**

**Running title:** Uncontrolled hypertension and mortality

**Authors:**

Donghao Zhou, *MD*^1^ Bo Xi, *MD*^2*^, Min Zhao, *MD*^3^ Liang Wang, *MD*^4^ Sreenivas P Veeranki, *MD* ^5^

**Affiliations:**

1. Department of Endocrinology, Linyi People's Hospital, 276003, Linyi, China
2. Department of Epidemiology, School of Public Health, Shandong University, Jinan, China
3. Department of Nutrition and Food Hygiene, School of Public Health, Shandong University, Jinan, China
4. Department of Biostatistics and Epidemiology, College of Public Health, East Tennessee State University, Johnson City, Tennessee, USA
5. Department of Preventive Medicine and Community Health, University of Texas Medical Branch, Galveston, Texas, USA

## ^*^ Correspondence to: Bo Xi, 44 Wenhuaxi Road, Department of Epidemiology, School of Public Health, Shandong University, Jinan 250012, China.

## Fax/Tel: +86-531-88382141. Email: [xibo2007@126.com](mailto:xibo2007@126.com)

| **Supplemental Table 1. Association of hypertension status with all-cause and CVD mortality by sex, age group and race/ethnicity** | | | | | | | | | | | | |
| --- | --- | --- | --- | --- | --- | --- | --- | --- | --- | --- | --- | --- |
| Hypertension status |  | All causes |  |  | CVD |  |  | Heart disease |  |  | Cerebrovascular diseases |  |
|  | n/ N | HR (95% CI) | *p* value | n/N | HR (95% CI) | *p* value | n/N | HR (95% CI) | *p* value | n/N | HR (95% CI) | *p* value |
| **Sex** |  |  |  |  |  |  |  |  |  |  |  |  |
| **Men** |  |  |  |  |  |  |  |  |  |  |  |  |
| Normal | 891/4886 | 1.00 (Reference) |  | 201/4886 | 1.00 (Reference) |  | 168/4886 | 1.00 (Reference) |  | 33/4886 | 1.00 (Reference) |  |
| Treated and controlled | 137/274 | 1.20 (0.92-1.57) | 0.168 | 35/274 | 1.36 (0.83-2.21) | 0.215 | 28/274 | 1.30 (0.77-2.21) | 0.317 | 7/274 | 1.77 (0.55-5.64) | 0.330 |
| Treated but uncontrolled | 253/396 | 1.55 (1.19-2.01) | 0.001 | 95/396 | 1.89 (1.20-2.97) | 0.007 | 67/396 | 1.72 (1.03-2.87) | 0.039 | 28/396 | 3.05 (1.31-7.13) | 0.011 |
| Untreated | 540/1051 | 1.45 (1.23-1.72) | <0.001 | 184/1051 | 2.10 (1.47-3.01) | <0.001 | 139/1051 | 2.01 (1.34-3.03) | 0.001 | 45/1051 | 3.46 (1.54-7.77) | 0.003 |
| **Women** |  |  |  |  |  |  |  |  |  |  |  |  |
| Normal | 670/5414 | 1.00 (Reference) |  | 156/5414 | 1.00 (Reference) |  | 118/5414 | 1.00 (Reference) |  | 38/5414 | 1.00 (Reference) |  |
| Treated and controlled | 198/469 | 1.11 (0.84-1.47) | 0.453 | 56/469 | 0.95 (0.56-1.60) | 0.838 | 43/469 | 0.92 (0.51-1.66) | 0.769 | 13/469 | 1.02 (0.36-2.91) | 0.964 |
| Treated but uncontrolled | 382/593 | 1.63 (1.34-1.99) | <0.001 | 145/593 | 2.35 (1.71-3.23) | 0.000 | 102/593 | 2.46 (1.66-3.65) | <0.001 | 43/593 | 2.78 (1.43-5.39) | 0.003 |
| Untreated | 479/864 | 1.31 (1.06-1.61) | 0.012 | 155/864 | 1.39 (0.94-2.05) | 0.095 | 106/864 | 1.29 (0.81-2.08) | 0.278 | 49/864 | 1.82 (1.00-3.33) | 0.050 |
| **Age group** |  |  |  |  |  |  |  |  |  |  |  |  |
| **<60 years** |  |  |  |  |  |  |  |  |  |  |  |  |
| Normal | 696/8941 | 1.00 (Reference) |  | 104/8941 | 1.00 (Reference) |  | 83/8941 | 1.00 (Reference) |  | 21/8941 | 1.00 (Reference) |  |
| Treated and controlled | 77/354 | 1.41 (0.98-2.01) | 0.061 | 16/354 | 1.38 (0.79-2.43) | 0.253 | 11/354 | 1.25 (0.66-2.38) | 0.490 | 5/354 | 2.23 (0.68-7.27) | 0.179 |
| Treated but uncontrolled | 98/303 | 2.08 (1.33-3.27) | 0.002 | 29/303 | 3.66 (1.70 -7.86) | 0.001 | 19/303 | 3.47 (1.56 -7.69) | 0.003 | 10/303 | 5.43 (1.81-16.31) | 0.003 |
| Untreated | 205/860 | 1.57 (1.14-2.16) | 0.007 | 60/860 | 2.69 (1.43-5.09) | 0.003 | 44/860 | 2.76 (1.32-5.79) | 0.008 | 16/860 | 2.73 (0.86-8.63) | 0.086 |
| **≥60 years** |  |  |  |  |  |  |  |  |  |  |  |  |
| Normal | 865/1359 | 1.00 (Reference) |  | 253/1359 | 1.00 (Reference) |  | 203/1359 | 1.00 (Reference) |  | 50/1359 | 1.00 (Reference) |  |
| Treated and controlled | 258/389 | 1.04 (0.85-1.28) | 0.701 | 75/389 | 0.98 (0.64-1.48) | 0.906 | 60/389 | 0.96 (0.61-1.53) | 0.873 | 15/389 | 1.26 (0.47-3.36) | 0.636 |
| Treated but uncontrolled | 537/686 | 1.46 (1.25-1.71) | <0.001 | 211/686 | 1.85 (1.44-2.36) | <0.001 | 150/686 | 1.84 (1.39-2.43) | <0.001 | 61/686 | 2.52 (1.43-4.45) | 0.002 |
| Untreated | 814/1055 | 1.23 (1.09-1.38) | 0.001 | 279/1055 | 1.42 (1.10-1.83) | 0.008 | 201/1055 | 1.34 (0.98-1.82) | 0.063 | 78/1055 | 2.30 (1.38-3.81) | 0.002 |
| **Race/ethnicity** |  |  |  |  |  |  |  |  |  |  |  |  |
| **No****n-Hispanic white** |  |  |  |  |  |  |  |  |  |  |  |  |
| Normal | 735/3686 | 1.00 (Reference) |  | 184/3686 | 1.00 (Reference) |  | 147/3686 | 1.00 (Reference) |  | 37/3686 | 1.00 (Reference) |  |
| Treated and controlled | 186/352 | 1.11 (0.90-1.38) | 0.324 | 49/352 | 1.08 (0.71-1.64) | 0.730 | 42/352 | 1.12 (0.71-1.77) | 0.624 | 7/352 | 1.02 (0.34-3.07) | 0.978 |
| Treated but uncontrolled | 312/437 | 1.57 (1.26-1.95) | <0.001 | 120/437 | 2.13 (1.56-2.93) | <0.001 | 93/437 | 2.21 (1.52-3.20) | <0.001 | 27/437 | 2.37 (1.37-4.09) | 0.003 |
| Untreated | 514/828 | 1.35 (1.15-1.59) | 0.001 | 172/828 | 1.71 (1.25-2.33) | 0.001 | 132/828 | 1.72 (1.19-2.49) | 0.005 | 40/828 | 1.81 (1.08-3.04) | 0.026 |
| **Non-Hispanic black** |  |  |  |  |  |  |  |  |  |  |  |  |
| Normal | 387/2828 | 1.00 (Reference) |  | 387/2828 | 1.00 (Reference) |  | 57/2828 | 1.00 (Reference) |  | 12/2828 | 1.00 (Reference) |  |
| Treated and controlled | 102/263 | 1.11 (0.84-1.48) | 0.444 | 102/263 | 1.38 (0.78-2.46) | 0.264 | 21/263 | 1.09 (0.55-2.17) | 0.804 | 9/263 | 2.29 (0.75-6.98) | 0.139 |
| Treated but uncontrolled | 199/345 | 1.55 (1.26-1.91) | <0.001 | 199/345 | 2.19 (1.37-3.49) | 0.002 | 42/345 | 1.86 (1.05-3.30) | 0.033 | 27/345 | 3.96 (1.53-10.24) | 0.006 |
| Untreated | 260/554 | 1.53 (1.26-1.85) | <0.001 | 260/554 | 2.20 (1.35-3.58) | 0.002 | 59/554 | 2.25 (1.36-3.73) | 0.002 | 23/554 | 2.80 (1.14-6.88) | 0.026 |
| **Mexican-American** |  |  |  |  |  |  |  |  |  |  |  |  |
| Normal | 390/3300 | 1.00 (Reference) |  | 90/3300 | 1.00 (Reference) |  | 68/3300 | 1.00 (Reference) |  | 22/3300 | 1.00 (Reference) |  |
| Treated and controlled | 41/111 | 0.96 (0.70-1.31) | 0.800 | 11/111 | 1.01 (0.58-1.77) | 0.971 | 7/111 | 0.87 (0.43-1.76) | 0.695 | 4/111 | 1.18 (0.38 -3.69) | 0.772 |
| Treated but uncontrolled | 106/175 | 1.42 (1.00-2.01) | 0.050 | 44/175 | 1.86 (1.20-2.87) | 0.006 | 30/175 | 2.28 (1.32-3.95) | 0.004 | 14/175 | 5.23 (1.83-14.94) | 0.003 |
| Untreated | 224/474 | 1.44 (1.04-1.98) | 0.027 | 80/474 | 2.67 (1.63-4.39) | <0.001 | 52/474 | 1.37 (0.75-2.53) | 0.298 | 28/474 | 3.74 (1.78 -7.85) | 0.001 |
| Adjusted for sex, age, race/ethnicity, education level, smoking, alcohol intake, BMI, TC, cholesterol-lowering medication use and diagnosed diabetes | | | | | | | | | | | | |

| **Supplemental Table 2. Association of hypertension status with all-cause and CVD mortality among older adults (aged ≥60 years)** | | | | | | | | | | | | |
| --- | --- | --- | --- | --- | --- | --- | --- | --- | --- | --- | --- | --- |
| Hypertension status |  | All causes |  |  | CVD |  |  | Heart disease |  |  | Cerebrovascular diseases |  |
|  | n/ N | HR (95% CI) | *p* value | n/N | HR (95% CI) | *p* value | n/N | HR (95% CI) | *p* value | n/N | HR (95% CI) | *p* value |
| Normal (<150/90 mmHg) | 1145/1744 | 1.00 (Reference) |  | 338/1744 | 1.00 (Reference) |  | 260/1744 | 1.00 (Reference) |  | 78/1744 | 1.00 (Reference) |  |
| Treated and controlled (<150/90 mmHg) | 407/601 | 1.09 (0.91-1.30) | 0.337 | 131/601 | 1.12 (0.81-1.54) | 0.493 | 103/601 | 1.15 (0.79-1.67) | 0.449 | 28/601 | 1.11 (0.57-2.16) | 0.746 |
| Treated but uncontrolled (**≥**150/90 mmHg) | 388/474 | 1.51 (1.24-1.85) | <0.001 | 155/474 | 1.91 (1.39-2.64) | <0.001 | 107/474 | 1.95 (1.34-2.84) | 0.001 | 48/474 | 2.51 (1.36-4.63) | 0.004 |
| Untreated (**≥**150/90 mmHg) | 534/670 | 1.28 (1.12-1.46) | 0.001 | 194/670 | 1.47 (1.20-1.79) | <0.001 | 144/670 | 1.47 (1.13-1.89) | 0.004 | 50/670 | 2.15 (1.23-3.73) | 0.008 |
| Adjusted for sex, age, race/ethnicity, education level, smoking, alcohol intake, BMI, TC, cholesterol-lowering medication use and diagnosed diabetes | | | | | | | | | | | | |
